# Effect of Water Vapor and Surface Morphology on the Low Temperature Response of Metal Oxide Semiconductor Gas Sensors

**DOI:** 10.3390/ma8095323

**Published:** 2015-09-23

**Authors:** Konrad Maier, Andreas Helwig, Gerhard Müller, Pascal Hille, Martin Eickhoff

**Affiliations:** 1 Airbus Group Innovations, München D-81663, Germany; E-Mails: konrad.maier@airbus.com (K.M.); mueller.g.u.s.grafing@t-online.de (G.M.); 2 Fachbereich 06, Munich University of Applied Sciences, Lothstraße 34, Munich D-80335, Germany; 3 I. Physikalisches Institut, Justus-Liebig-Universität Gießen, Gießen 35392, Germany; E-Mails: pascal.hille@physik.uni-giessen.de (P.H.); eickhoff@exp1.physik.uni-giessen.de (M.E.)

**Keywords:** low temperature gas response, integrator gas response, SnO_2_, surface morphology, water vapor, BET adsorption

## Abstract

In this work the low temperature response of metal oxide semiconductor gas sensors is analyzed. Important characteristics of this low-temperature response are a pronounced selectivity to acid- and base-forming gases and a large disparity of response and recovery time constants which often leads to an integrator-type of gas response. We show that this kind of sensor performance is related to the trend of semiconductor gas sensors to adsorb water vapor in multi-layer form and that this ability is sensitively influenced by the surface morphology. In particular we show that surface roughness in the nanometer range enhances desorption of water from multi-layer adsorbates, enabling them to respond more swiftly to changes in the ambient humidity. Further experiments reveal that reactive gases, such as NO_2_ and NH_3_, which are easily absorbed in the water adsorbate layers, are more easily exchanged across the liquid/air interface when the humidity in the ambient air is high.

## 1. Introduction

Over the past decade an increasing number of reports have emerged, demonstrating that—in addition to their high-temperature response—metal oxide (MOx) gas sensors can also exhibit a low-temperature response that persists from room temperature up to the boiling point of water (100 °C) or slightly above. Gases that could be detected under such conditions are NO_2_, NH_3_, H_2_S and a number of short-chain alcohols [1,2,3,4,5,6,7]. In this latter mode of operation UV photo-activation has also been shown to be effective [8,9,10,11,12,13]. A second group of materials that exhibits a similar kind of low-temperature response, is III-nitride materials both in the form of AlGaN/GaN high electron mobility transistors (HEMT) with flat surface morphologies [14,15,16,17,18,19] as well as in the form of GaN/InGaN nanowire heterostructures [20,21,22]. These effects can be attributed to the fact that III-nitride surfaces tend to develop surface oxides when exposed to ambient air which makes them similar to conventional MOx materials [23,24,25,26,27,28]. A third group of materials that exhibits a well-documented low low-temperature gas response is hydrogenated diamond. There, a selective response to acid and base forming gases can be observed which often takes the form of an integrator-like gas response [29,30,31,32,33]. Response characteristics of this latter kind can be observed on diamond materials with surface morphologies ranging all the way from nano- up to single-crystalline [29,30,31,32,33,34,35,36,37,38,39,40]. The interesting aspect with regard to this latter group is that among the diamond community there seems to be a general consensus that this low-temperature response is mediated by a thin layer of physisorbed water and that this response can be explained on the basis of the so-called transfer doping model [41]. In essence, this model states that gas absorption in the physisorbed water layer leads to pH changes in the adsorbate layer and that these changes, in turn, are communicated to the charge carrier densities in the underlying semiconductor substrates. An overall look at these results shows that a low temperature response to NO_2_—and to a lesser extent to NH_3_—seems to be a fairly universal trend stretching over different kinds of semiconductor materials [42]. The integrator-type response, however, seems to be a much less universally observable phenomenon and likely appears only when certain morphological boundary conditions are satisfied. Indications into this latter direction are that nanowire gas sensors exhibit a humidity response which is proportional to the humidity in the gas ambient [43,44] whereas materials with flat surface morphologies seem to be much less able to follow changes in the ambient humidity [7,29,45].

In the following, we shed more light on the ability of semiconductor substrates of acquiring multi-layer water adsorbates and of exhibiting an integrator-type gas response with regard to acid- and base-forming gases. Below, we will demonstrate that Brunauer–Emmett–Teller (BET) water adsorbates [46,47] can readily form on MOx surfaces with a wide range of different surface morphologies. In contrast, the rates of adsorption and, even more, desorption of BET water layers are shown to depend on the surface morphology: while rough surface morphologies enhance the speed with which BET adsorbates can respond to changes in the ambient humidity, flat surface morphologies tend to stabilize BET adsorbates, particularly under conditions of low ambient humidity. Once BET adsorbates have been formed, the rates of exchange of acid- and base-forming gases across the liquid–air interface are strongly humidity-dependent: whereas acid- and base-formers remain almost permanently trapped inside the BET adsorbates at low humidity levels, high humidity levels in the ambient air allow the reactive gas concentrations in the air and liquid phases to be rapidly equilibrated.

## 2. Results and Discussion

### 2.1. Sensing Layers and Surface Morphology

**Figure 1:**
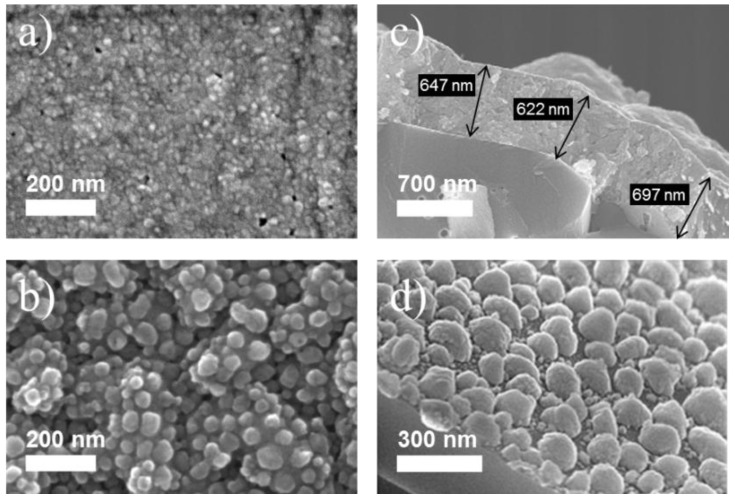
Surface morphologies of a flat-surface (**a**,**c**) and of a granular surface morphology film (**b**,**d**). Images (**a**) and (**b**) present top surface scanning electron microscopy (SEM) pictures of both kinds of films; images (**c**) and (**d**) display similar films after cleaving their substrates and after performing oblique-angle SEM on the cleaving edges.

In order to test the effect of the surface morphology on the room temperature gas response, we have adopted two different methods of preparing SnO_2_ layers with distinctly different and well-controllable surface morphologies. Typical morphologies obtained with both methods are shown in Figure 1. The top row of this figure shows the morphology of films produced by electron beam evaporation of SnO_2_ at room temperature and high-temperature annealing in air. As shown in the surface SEM of Figure 1a, this method produces compact films consisting of many small crystallites with diameters in the 10 nm range. The cross sectional SEM obtained on a similar film after cleaving the substrate (Figure 1c) shows that these films are very compact, exhibiting very little porosity. Furthermore, on a micrometer scale, their surface topography is seen to be essentially flat, following more or less the roughness of the substrates. The bottom row of Figure 1 shows a film with a very rich surface morphology with typical feature sizes in the range of ten to several tens of nano-meters (Figure 1b). The oblique-angle SEM image in Figure 1d shows that the morphology of these latter films is very different from the first ones, consisting of an array of fully developed 3-dimensionsional SnO_2_ grains. Almost all of these grains have a visible sub-structure with feature sizes in the range of a few nanometers only. In addition, a larger number of such smaller grains can be seen in the empty spaces between the dominating larger grains. These latter films were produced by a modified RGTO (rheotaxial growth and thermal oxidation) technique, starting with a room-temperature evaporation of metallic tin and a high-temperature annealing step in air to allow the molten tin to become oxidized and to be transformed into SnO_2_. A more detailed description of the preparation processes and the resulting material properties is presented in Section 3. In the following our main focus is on the gas sensing properties of such films, and in particular, on their interaction with the humidity in the ambient air.

### 2.2. Gas Sensing Properties

In order to assess the gas sensing properties of the above layers, the films were fitted with contact pads and gas sensing tests with humidity, NO_2_, NH_3_, H_2_, C_2_H_4_, CO, and ethyl alcohol were performed. All gases were diluted to the desired concentrations in dry synthetic air. The sensor operation temperature could be controlled with the help of a substrate heater, allowing measurements between room temperature and about 100 °C. In order to minimize parasitic condensation on the chamber walls, the walls were slightly heated (~40 °C) during all gas sensing tests. The actual humidity concentration inside the test chamber was independently controlled using a humidity sensor.

#### 2.2.1. Humidity Response

**Figure 2:**
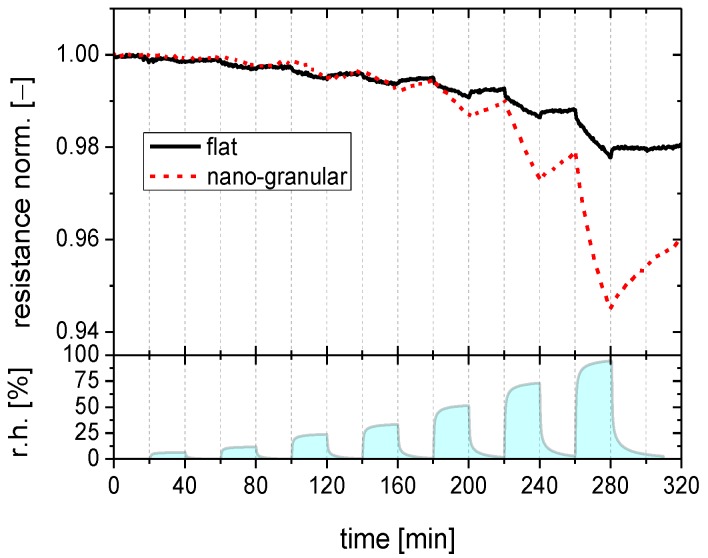
Gas response of SnO_2_ layers with a flat (full line) and a nano-granular (broken line) surface morphology towards water vapor when stored close to room temperature.

Before starting gas sensing tests, both kinds of sensing materials were heated and stored in a flow of dry synthetic air to remove any humidity that might have existed on the sensor surface. Thereafter the films were cooled to nominally room temperature and subjected to pulses of humidified synthetic air with the level of humidity increasing in each subsequent exposure pulse. Between each exposure pulse the initially dry flow of synthetic air was restored. The data displayed in Figure 2 show that SnO_2_, both in its flat-surface and nano-morphology versions, does respond to water vapor with the expected resistivity decrease. The magnitude and the dynamic behavior of the humidity responses, however, clearly depend on the surface morphology. The morphologies of the tested films are those shown in Figure 1a,b above.

The data in Figure 2 show that the SnO_2_ film with a smooth surface exhibits an almost ideal kind of integrator response with the humidity response increasing linearly during each exposure pulse and with the response staying constant in between exposure pulses. From the fact that there is no visible drop in the sensor response over periods ranging up to 40 min, it is evident that the time constants for desorption from room temperature substrates is in the order of several hours at least. Due to this extremely slow desorption, the flat-surface films accumulate increasing amounts of water as the number of humidity exposure pulses is increased. In the nano-morphology case a different behavior is observed: the humidity response also rises linearly during each exposure pulse. After each exposure pulse, however, the humidity response drops much more rapidly than in the flat-surface case. Desorption time constants in this case were in the order of one hour only.

**Figure 3:**
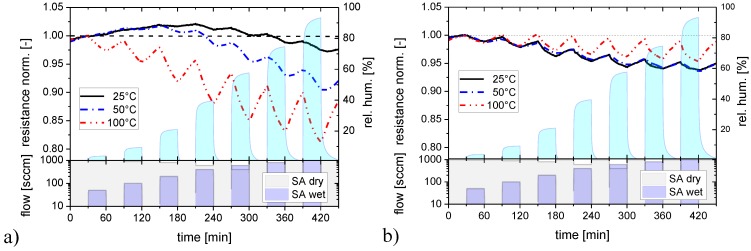
Response of SnO_2_ layers with a flat (**a**) and a nano-granular (**b**) surface morphology towards water vapor at different sensor operation temperatures.

These first experiments show that adsorbed water vapor tends to stick to SnO_2_ surfaces and that the degree of sticking depends on the surface morphology. Since besides morphology, temperature is an important parameter controlling the adsorption/desorption kinetics, these experiments were repeated at increasingly higher sensor operation temperatures, as reported in Figure 3 with the data for flat-surface (Figure 3a) and nano-granular films (Figure 3b) displayed in separate diagrams. For flat-surface films the adsorption and desorption rates increase as the substrate temperature is raised. The increase in the adsorption rates allows increasingly larger sensor responses to be built up during each exposure pulse as the temperature is raised. The concomitant increase in desorption rates causes the response between the humidity exposure pulses to drop more rapidly. This latter effect, however, never increases to the extent that a full recovery towards baseline can be reached between two humidity exposure pulses. As a consequence, flat-surface films still retain traces of integrator behavior at the highest temperatures investigated. In the nano-granular case, similar observations were made. However, higher adsorption and desorption rates were observed at all substrate temperatures. As a consequence, the integration behavior almost disappears as the sensor operation temperature is raised to 100 °C.

#### 2.2.2. Response to Acid- and Base-Forming Gases

**Figure 4:**
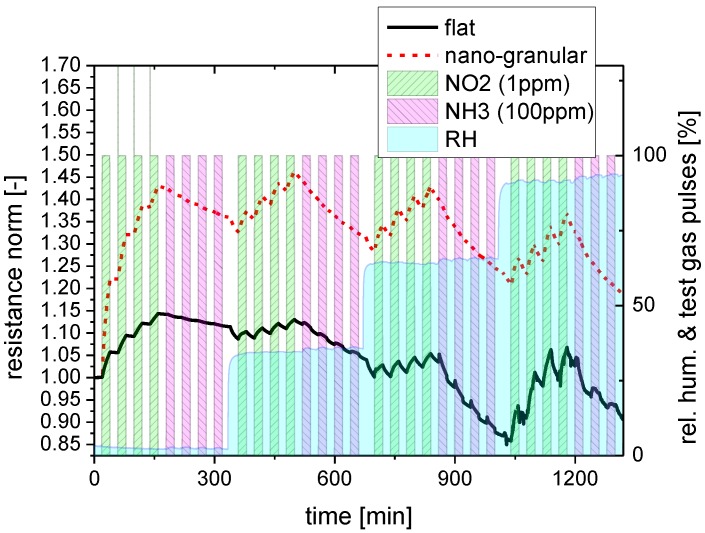
Response of SnO_2_ layers with a flat (red) and a nano-granular (black) surface morphology towards NO_2_ and NH_3_ exposure pulses in different humidity backgrounds (rh).

Both kinds of sensing materials were subjected to a series of NO_2_ and NH_3_ exposure steps to investigate their response towards water-soluble gases. In order to elucidate the effects of background humidity this experiment was repeated four times, each time increasing the humidity level of the synthetic air carrier gas from almost zero up to about 90% relative humidity. Figure 4 illustrates the timing of these exposure sequences in more detail, showing at the same time the reaction of the flat-surface and nano-granular SnO_2_ films to these exposure sequences. For better comparison, the sensor resistances of the two versions of SnO_2_ have been normalized to their starting values at the beginning of these sensing tests.

As a key result one can observe that both kinds of SnO_2_ do respond to these acid- and base-forming gases. Considering the absolute magnitude of the gas response it is seen that SnO_2_ layers with nano-crystal morphology exhibit a larger response than their flat-surface counterparts when the background humidity level is low. In the limit of high-humidity levels the magnitude of the gas response becomes more or less similar and independent of the surface morphology. Turning to the kinetics of the gas response, it is seen that both morphologies exhibit a more or less pronounced integrator-type response as long as the background humidity is low and a faster and more reversible response when the background humidity is high. This latter result demonstrates that the presence of water vapor does have an accelerating effect on the desorption behavior of NO_2_ and NH_3_ molecules from the adsorbed water layers, independent of the kind of surface morphology. Comparing NO_2_ and NH_3_, it is seen that the adsorption and desorption rates of NO_2_ are relatively high, whereas they are very low in the case of NH_3_. In this latter case the accelerating effect of the background humidity on the adsorption and desorption processes is much more important than in the case of NO_2_. In order to reveal any interaction of the dissolved NO_2_ and NH_3_ molecules inside the water adsorbate layers, control experiments were performed in which the sequence of the NO_2_ and NH_3_ exposure steps was inverted. As these latter experiments yielded similar results, it appeared that the adsorbates do not measurably react inside the adsorbed water layers and that therefore only their relative concentrations do determine the actual level of gas response.

#### 2.2.3. Response to Non-Acid and Non-Base Forming Gases

**Figure 5:**
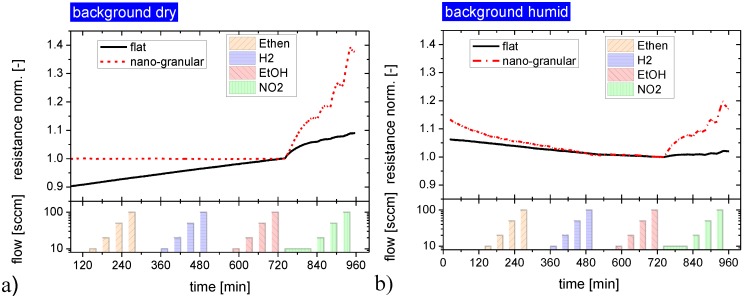
Gas response of SnO_2_ layers with a flat and a nano-granular surface morphology towards non-acid and non-base forming gases. The response to the acid former NO_2_ is shown for comparison at the end of each exposure sequence; background dry synthetic air (**a**) and humidified synthetic air background, rh = 30% (**b**).

In order to gain further insight into the gas sensing mechanisms operative on both kinds of materials, similar gas sensing tests were performed with the samples being exposed to increasing concentrations of non-acid or non-base-forming gases. These latter data, displayed in Figure 5, clearly prove that there is no response towards non-acid and non-base-forming gases, independent of the surface morphology of the SnO_2_ layers. In addition the response to this latter group of gases also did not depend on the level of background humidity. Again, and consistent with the results in Figure 4, flat-surface films exhibited a lower NO_2_ response than those with a granular surface.

### 2.3. Formation of BET Adsorbates

Overall, the observed gas response characteristics of the SnO_2 _layers are very similar to those observed on hydrogenated diamond samples [29,30,31,32,33,34,35,36,37,38,39,40]. Considering the fact that roughly the same gas sensing characteristics are observed on materials with drastically different surface properties, it is strongly suggested that the basic sensing mechanism is related to the adsorbed water layers, rather than to any direct interaction of the analytes with the different solid surfaces themselves. Further considering that the transfer model of doping [41] can satisfactorily explain the gas sensing phenomena on hydrogenated diamond materials; we should like to consider here in more detail the possibility of multi-layer BET adsorption of water vapor on metal oxide sensor surfaces and thus the possible existence of similar transfer processes on metal oxide surfaces. Water vapor, unlike most other gases of interest in gas sensing applications, has an abnormally high melting and boiling point. Water vapor therefore may undergo multi-layer physisorption on a sensor surface, particularly on substrates which are slightly cooler than the gas ambient [46,47,48]. In the water vapor sensing experiments described above the sensor substrate is isothermal with the surrounding gas phase at a temperature T close to room temperature. In this special case the water vapor pressure in the gas phase can be expressed as $P\left(T\right)=rh\;P_{sat}\left(T\right)$ with rh standing for the relative humidity in the gas ambient and $P_{sat}\left(T\right)$ for the saturated water vapor pressure at this temperature. Under these special conditions the BET isotherm relates the surface coverage with water, θ, to the relative humidity in the gas phase via:

(1) $$\text{θ}\left(rh,T\right)=\frac{rh}{1-rh}\;\frac{b_{BET}\left(T\right)}{\left[1+rh\;\left(b_{BET}\left(T\right)-1\right)\right]}$$

with *b_BET_*(*T*) standing for the so-called BET constant:

(2) $$b_{BET}\left(T\right)=exp\left[\frac{\varepsilon_{1}-{\text{ε}}_{lf}}{k_{B}T}\right]$$

**Figure 6:**
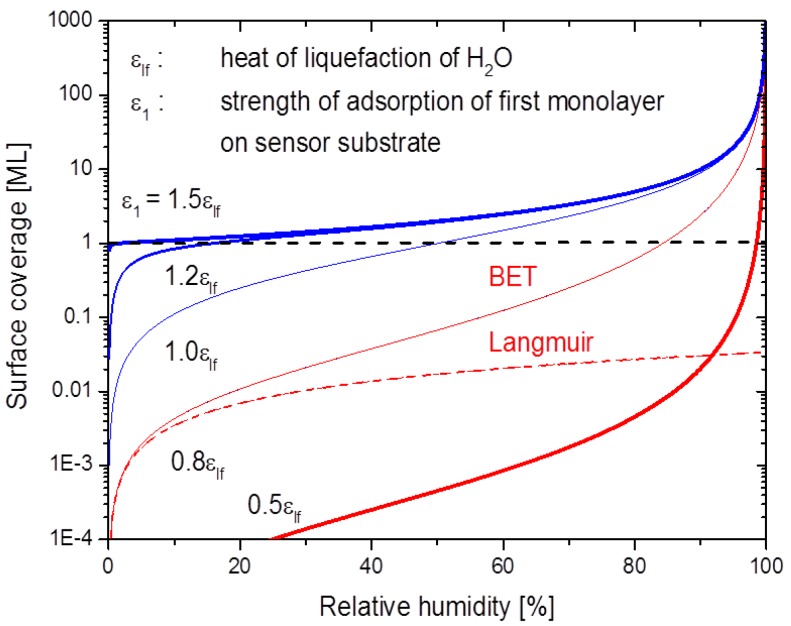
BET coverage in monolayers (ML) at 293 K as a function of the relative humidity. The degree of BET adsorption critically depends on the strength of adsorption ${\text{ε}}_{1}$ of the first monolayer on the substrate. The full and broken lines at ${\text{ε}}_{lf}$ = 0.8 show that Langmuir adsorption isotherms approximate BET ones in the limit of low humidity.

The two energy parameters in this latter equation, ${\text{ε}}_{1}$ and ${\text{ε}}_{lf}$, measure the strength of adsorption of the first monolayer directly on the substrate itself (${\text{ε}}_{1}$) and ${\text{ε}}_{lf}$ the strength of adsorption in each following monolayer of water molecules. Quantitatively, $\varepsilon_{lf}$ is the heat of liquefaction of bulk water ${\text{ε}}_{lf}$ ~ 0.445 eV per molecule) [46]. A numerical evaluation of the above equations demonstrates that the parameter $\varepsilon_{1}$ plays a key role in enabling or disenabling BET multilayer absorption on a sensor surface. The data displayed in Figure 6 demonstrate that multilayer adsorption can readily occur when the binding of H_2_O molecules to the adsorbent surface is stronger than the binding between water molecules in liquid water $\epsilon_{1}>\;{\text{ε}}_{lf}$) and virtually impossible otherwise. Inspection of the above equations also shows that in the limit of low humidity (rh < 0.1), BET isotherms are well approximated by the more familiar Langmuir isotherms:

(3) $$\text{θ}\left(\text{rh},\text{T}\right)=\frac{\text{rh}}{\text{rh}+1/{\text{b}}_{\text{BET}}\left(\text{T}\right)}$$

This latter fact is also illustrated in Figure 6.

### 2.4. Adsorption and Desorption Kinetics

The considerations in Figure 6 are useful for demonstrating the plausibility of BET adsorption on room-temperature sensor substrates. In order to make contact with our experimental data, the kinetics of the Langmuir and BET adsorption processes need to be considered in more detail. While the description of the Langmuir adsorption kinetics is trivial, the modeling of BET adsorption processes requires the numerical integration of a set of coupled differential equations as described in more detail in the appendix. Here, we turn to those results that have been obtained by fitting Langmuir and BET adsorption isotherms to the humidity response data in Figure 2.

**Figure 7:**
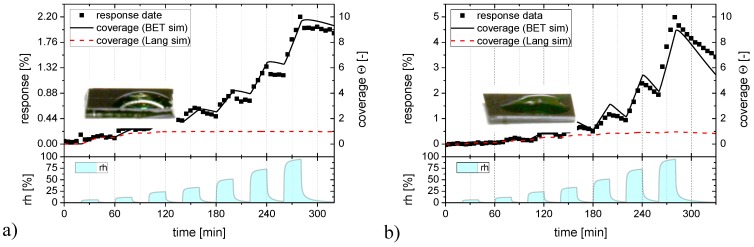
Langmuir (red) and BET (black) adsorption/desorption processes fitted to the humidity response (full squares) of the flat-surface SnO_2_ layer (**a**) and to a SnO_2_ layer with nano-granular morphology (**b**).

In Figure 7 the humidity response data of Figure 2 are reproduced as black solid squares and the Langmuir and BET fits as full colored lines. Turning to Figure 7a first, it is seen that a Langmuir adsorption process (red line) can well approximate the low humidity part of the flat-surface sensor data but that it fails to do so at the higher humidity levels. A BET process (black line) obviously provides a much better fit over the whole range of humidity levels. We can take this as evidence that SnO_2_ surfaces are reasonably hydrophilic and that therefore BET multilayer adsorbates do indeed form. Supporting evidence into this direction is provided by the fact that the contact angle of water is small on the investigated sensor surfaces (insets in Figure 7) and from the theoretical results of Figure 6, which show that monolayer adsorbates should already arise at extremely low levels of humidity when the H_2_O binding on the adsorbent surface is stronger than the hydrogen-bridge bonding in liquid water. Figure 7b repeats the humidity response data of Figure 2 for the granular morphology layers, plotting at the same time the corresponding Langmuir and BET fits. Again, a BET process provides a much better fit over the entire range of humidities than a Langmuir one.

The most interesting items that can be extracted from such fits are kinetic parameters for adsorption and desorption for the different types of SnO_2_. For ease of comparison these data are listed in Table 1. Turning to the Langmuir parameters first, information can be obtained with regard to the adsorption and desorption on the semiconductor substrates themselves. Comparing the data sets for the flat-surface and nano-morphology cases, it is seen that the adsorption constant $k_{ads}$ attains fairly similar values on both kinds of substrates, whereas $k_{des}$ is enhanced by roughly a factor of two when the desorption occurs from a nano-granular surface.

In the BET case, the parameters α and β take on almost the same values as the Langmuir ones $k_{ads}$ and $k_{des}$ as these measure the rates of adsorption and desorption directly on the adsorbent surface. The interesting new piece of information is contained in the BET parameters γ and δ, which measure the rates of adsorption and desorption at pre-adsorbed water layers. Comparing these latter parameters to the substrate-specific α and β ones, a somewhat weaker adsorption on pre-adsorbed water layers is inferred and a considerably enhanced desorption from such layers. Similar to the Langmuir case, the rate of desorption of BET-adsorbed water is roughly a factor of two higher from nano-granular as opposed from flat surfaces.

**Table 1:** Kinetic parameters for Langmuir and BET adsorption and desorption processes obtained from the data fits in Figure 6.

Process	Kinetic Parameter	Flat Surface	Nano-Granular
Langmuir adsorption	*k_ads_*	1 × 10^−4^	1 × 10^−4^
Langmuir desorption	*k_des_*	0.2 *k_ads_*	0.2 *k_ads_*
BET adsorption on substrate	α	1 × 10^−4^	1 × 10^−4^
BET desorption from substrate	β	0.2 α	0.35 α
BET adsorption onto pre-adsorbed H_2_O	γ	0.35 α	0.5 α
BET desorption from pre-adsorbed H_2_O	δ	8.75 α	15 α

Theoretically, desorption enhancements of H_2_O on nano-structured surfaces are expected on the basis of the Kelvin equation [49,50,51]:

(4) $$P_{sp}\left(T,r\right)=P_{H2\text{O}}\left(T\right)\;exp\left[\frac{2\;v_{H2\text{O}}\;{\text{σ}}_{H2\text{O}}}{k_{B}\;T\;r}\right]$$

This latter equation states that the vapor pressure over a sphere of radius r is enhanced relative to the equilibrium vapor pressure $P_{H2\text{O}}\left(T\right)\;$ over a flat surface kept at the same temperature T. The determining parameters in this equation are the radius of curvature *r* of the sphere, the molar volume of water $v_{H2\text{O}}$ and the surface tension ${\text{σ}}_{H2\text{O}}$. In essence this latter quantity measures the amount of energy that needs to be expended to move a molecule of water from the bulk of a volume of liquid water to its surface. For a water-air interface this quantity amounts to roughly 0.1 eV/molecule [49]. This amount is positive as a H_2_O molecule at a water-air interface can form fewer hydrogen bonds than one in the liquid bulk.

**Figure 8:**
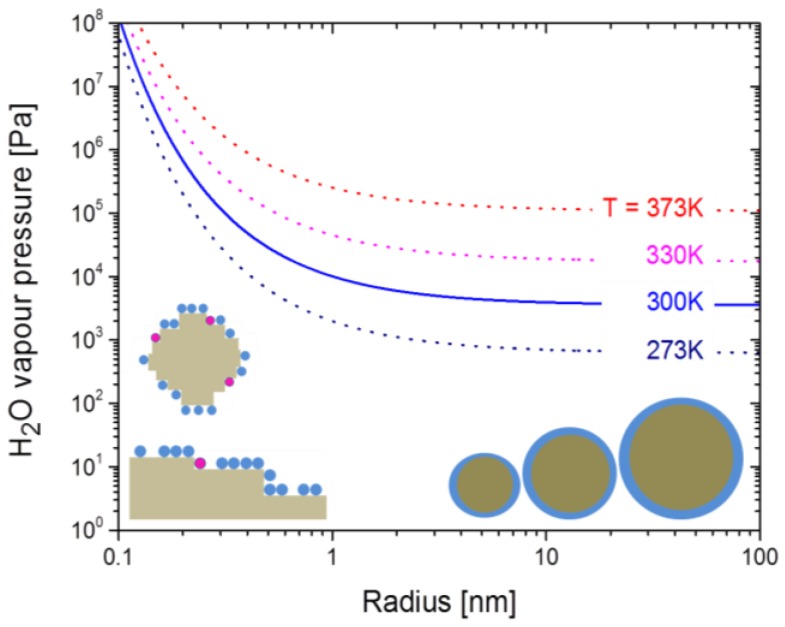
Vapor pressure enhancement over small spheres with radius *r* relative to a flat water-air interface as predicted by the Kelvin equation [51]. The spheres in the bottom right-hand part indicate water desorption from ideal spheres as considered by the Kelvin equation; the pictures in the left-hand part indicate that real, nanometer-sized crystals will have step-, kink- and terrace-kinds of defects of sub-nanometer size.

Figure 8 displays the variation of the water vapor pressure as a function of the sphere radius as predicted by the Kelvin equation. These data clearly show that sizeable desorption enhancements should only occur at morphological feature sizes in the order of 1 nm or below. In comparison, the morphological feature sizes that dominate the SEM images in Figure 1 are one to two orders of magnitude larger. These dominating features therefore are far too large to account for the observed desorption enhancements on nano-granular surfaces. Morphological features, at least one order or magnitude smaller than those visible in the SEM pictures, are kink sites at nano-granular surfaces and mono-atomic steps at the surface of seemingly flat-surface films. As such sub-nanometer-sized flaws should develop in much larger numbers on nano-meter-sized spheres than on flat surfaces, desorption enhancements on granular surfaces are expected. Consistent with this explanation the BET adsorption rates α and γ attain similar values on both kinds of surface morphologies, while the desorption parameters β and δ are consistently enhanced when nano-granular morphologies are involved.

Another interesting aspect of kink sites and step lines concerns the sensing of NO_2_ and NH_3_ molecules. Once such sites get uncovered by H_2_O, NO_2_ and NH_3_ molecules may directly interact with the underlying sensor substrates. The ongoing availability of H_2_O close to these adsorption sites enables these adsorbates to interact not only with the substrates themselves but also with water molecules diffusing across those sites. In this way a competition arises between reactive-gas/substrate and reactive-gas/water interactions. The data in Figure 4 indicate that such a competition might be advantageous in enhancing the response to such reactive gases. Recently, a similar process has also been claimed to be operative on porous graphene layers. There it was argued that the dipolar interaction of water molecules with adsorbed NO_2_ and NH_3_ molecules would help to detach these reactive analytes from graphene adsorption sites [52].

## 3. Experimental Section

In order to test the effect of the surface morphology on the room temperature gas response, we have adopted two different methods of preparing SnO_2_ layers with distinctly different and well-controllable surface morphologies. SnO_2_ layers with a flat surface morphology were prepared by physical vapor deposition (PVD) of SnO_2_ powders. Electron beam evaporation onto room temperature substrates and under standard vacuum conditions yielded amorphous material with an excess amount of tin. In order to arrive at fully stoichiometric, crystalline SnO_2_ layers, the evaporated films were annealed in laboratory air at 600 °C for 20 h [53]. X-ray analysis on the so-formed films confirmed that these consist of stoichiometric SnO_2_. Scanning electron microscopy further revealed that these films have a comparatively flat surface, are compact and do not exhibit any significant porosity. This latter fact is demonstrated in Figure 1a,c. Films with a granular morphology were prepared using a modified RGTO process [54]. These latter films were prepared by electron beam evaporation of Sn on silicon substrates with a 500 nm thick thermal SiO_2_ layer on top. In order to obtain metallic Sn layers with a flat surface morphology, the substrates were not heated during deposition. Oxidation of the evaporated tin layers was performed afterwards by annealing at 600 °C in laboratory air. Upon annealing, the evaporated tin layers melt ($T_{m}$ = 232 °C), forming small droplets which become oxidized as annealing proceeds. In order to obtain fully oxidized material, the oxidation time was increased in proportion with the thickness of the evaporated tin layer (~1 h for every 10 nm of evaporated Sn thickness) [53].

Oxidation of very thin Sn layers (d < 80 nm) yielded layers, consisting of monolayers of disconnected SnO*_x_* grains as shown in Figure 1d. Average diameters of the so-formed SnO*_x_* grains scaled with the thickness of the Sn precursor films. As shown in the example of Figure 1d, a SnO*_x_* layer formed from a 40 nm Sn precursor film consisted of an irregular array of SnO*_x_* grains with an average diameter around 40 nm. In addition, one can observe a large number of very small grains interspersed in between the larger ones. Such films did not electrically conduct as the disconnected grains failed to form percolation paths that could connect the contact pads at the periphery of the SnO_x_ samples. In order to obtain electrically conducting layers, a second precursor layer of Sn was evaporated on top of the first layer of SnO*_x_* grains and annealed in air as before. These second and third layers of SnO*_x_* grains formed bridges between the initially disconnected grains and thus allowed electrical currents to flow over macroscopic distances. A typical example of such an interconnected film is shown in Figure 1b.

XRD spectra on such films indicated that the formed spheres consisted mostly of the Cassiterit modification of SnO_2_ and a small amount of SnO. Average XRD grain sizes were in the order of 15 nm and thus significantly smaller than those grains that dominated the SEM pictures. The smaller XRD grain sizes indicate that the optically visible grains are agglomerates of much larger numbers of small grains. Further, as the annealing in air had been carried out for a sufficiently long time to arrive at fully oxidized material, the presence of SnO points to a certain degree of restructuring at the grain surfaces. It is likely that such areas of non-stoichiometry arise at kink sites and mono-atomic steps which form as small crystallites cannot form ideal spheres.

## 4. Conclusions

The room-temperature response of SnO_2_ gas sensing layers has been investigated. Our results indicate that in this low-temperature regime, water vapor can readily form multi-layer BET adsorbates with thicknesses in the range between one to ten monolayers. Once formed, these BET adsorbates can readily absorb reactive gases, which can easily undergo electrolytic dissociation in the adsorbed water films (e.g., NO_2_, NH_3_). The resulting pH changes in the BET layers are then communicated to the sub-surface electronic system inside the MOx semiconductors, thus forming an electrically detectable output signal. A huge variety of combustible gases (H_2_, simple hydrocarbons), which yield large signals on heated MOx gas sensors, are either completely rejected by the BET water layers (e.g., H_2_, C_2_H_4_) or fail to electrolytically dissociate (e.g., C_2_H_5_OH) and thus go undetected. BET adsorbates thereby considerably contribute to gas selectivity. Concerning the kinetics of those gases that actively contribute to the sensor response, it is found that the desorption of H_2_O, NO_2_ and NH_3_ is much more sensitively influenced by the effects of surface morphology, ambient humidity and sensor operation temperature than their adsorption. While rough surface morphologies, high ambient humidity and elevated sensor operation temperatures enhance desorption, desorption becomes increasingly unlikely in the limit of flat surface morphologies, low ambient humidity and room-temperature operation. In this latter case MOx gas sensors tend to function as gas dosimeters. As has been shown in recent papers [31,32,33], a reliable operation of dosimeter-type sensors is only possible, in the case that enforced periodic resets to sensor baseline are carried out. In the case that such resets are not applied, low-temperature MOx gas sensors appear to drift in unreliable and unpredictable manners. It is possibly for this lack of insight that the realm of low-temperature operated gas sensors has not been systematically investigated yet and that dosimeter-type sensors have not yet been extensively used in practical sensor applications.

## Figures and Tables

**Figure A1:**
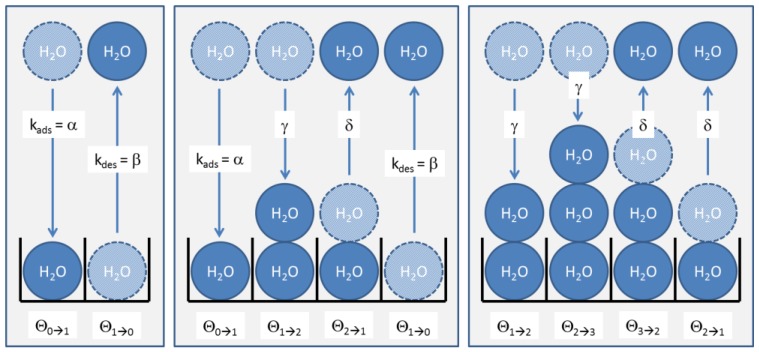
Formation of multi-layer BET adsorbates on a sensor surface. The three boxes indicate adsorption-/desorption processes occurring with a bare substrate, with a substrate covered with one monolayer and with two monolayers of H_2_O molecules.

## Appendix: Kinetics of BET Multi-Layer Adsorption

In order to fit the numerical models of adsorption according to Langmuir and BET kinetics, respectively, it is assumed that the gas response *S* of the MOx material is proportional to the number density of H_2_O molecules adsorbed on the surface:

(A1) $$S\left(N_{H2\text{O}}\right)=\frac{R_{0}-R}{R_{0}}\propto \;N_{H2\text{O}}$$

This equation can be justified on the assumption that the SnO_2_ sensing layers assume the form of thin-film resistors with an un-depleted layer thickness d and that the adsorption of H_2_O displaces negative surface oxygen ions, thus increasing the electrically effective layer thickness of these sensor from d to d+x. In this case:

(A2) $$S\left(x\right)\approx \;\frac{x}{d}$$

Further, assuming that the areal density of displaced surface oxygen ions is proportional (f) to the areal density of adsorbed H_2_O molecules, an equivalent density of positive bulk donors $N_{d}\;$ needs to disappear:

(A3) $$f\;N_{H2\text{O}}=x\;N_{d}$$

With the resulting change in the depletion layer thickness x, one finally obtains:

(A4) $$S\left(N_{H2\text{O}}\right)=\;\frac{f}{d}\frac{N_{H2\text{O}}}{N_{d}}$$

In the following two sections we show how the values of $N_{H2O}$ have been evaluated for the two scenarios of Langmuir monolayer and BET multi-layer adsorption.

### A.1. Langmuir Kinetics

Langmuir adsorption assumes a reversible reaction between adsorbate molecules in a gas phase A and adsorption sites S on an adsorbate solid leading to adsorbates SA:

(A5) $$\text{A}+\text{S}\underset{k_{des},\;k_{ads}\;}{↔}\;\text{SA}$$

With the adsorbate molecules only being able to interact with the adsorbent surface and not with each other, the maximum surface coverage is limited to one monolayer.

The kinetics of this process is described by the following rate equation [47,48]:

(A6) $$\frac{d\;N_{1}\left(t\right)}{dt}=k_{ads}\;{\text{p}}_{A}\left(t\right)\left(N_{SA}-N_{1}\left(t\right)\right)-k_{des}N_{1}\left(t\right)$$

where:

*N_1_(t)* … aerial density of adsorption sites occcupied with a single adsorbate at time *t*;

*N_SA_* … total density of adsorption sites on the surface;

*p_A_* … partial pressure in the gas phase;

*k_ads_* … adsorption rate;

*k_des_* … desorption rate.

Depending on the partial pressure ${\text{p}}_{A}$ and the magnitudes of the adsorption and desorption rate constants $k_{ads}$, and $k_{des}$, respectively, a fraction ${\text{θ}}_{1}$ of the available adsorption sites will be occupied with a single adsorbate, while the remaining fraction ${\text{θ}}_{0}=1-\;{\text{θ}}_{1}$ will be empty. In terms of surface coverages, Equation (A2) can be re-written as:

(A7) $$\frac{d{\text{θ}}_{1}\left(t\right)}{dt}=\;k_{ads}\;{\text{p}}_{A}\left(t\right)\left(1-{\text{θ}}_{1}\left(t\right)\right)-k_{des}{\text{θ}}_{1}\left(t\right)=k_{ads}\;{\text{p}}_{A}\left(t\right){\text{θ}}_{0}\left(t\right)-k_{des}{\text{θ}}_{1}\left(t\right)$$

### A.2. BET Kinetics

BET adsorption allows building multilayer adsorbate layers [46,47,48]. Similar to the Langmuir case, gas phase molecules A are able to directly adsorb on the adsorbent surface with rate constant $\text{α}=\;k_{ads}$ and to desorb from there with rate constant $\;\text{β}=\;k_{des}$. Additionally, and in contrast to the Langmuir case, gas phase molecules A are able to interact with molecules A already adsorbed on the adsorbent surface and to form on-top layers of adsorbed molecules A. The rate constants of adsorption and desorption in all subsequent on-top layers are assumed to be γ and δ, respectively. In the specific example of A = H_2_O, considered here, on-top layer adsorbates can be easily formed via hydrogen bridges connecting an already adsorbed H_2_O molecule with the oxygen lone pair electrons of a H_2_O molecule in the nearest-neighbor on-top layer.

In this way multi-layer adsorbates can be built up as shown in Figure A1. In this graph the adsorbent surface is sub-divided into $N_{SA}\;$individual cells to indicate potential adsorption sites. Each of these is assumed to be able to accommodate 0, 1, 2,  …,  n H_2_O molecules. In such a multi-layer adsorption scenario the total coverage of adsorption sites with sites containing 0, 1, 2,  …,  n adsorbates is:

(A8) $$1={\text{θ}}_{0}+\;{\text{θ}}_{1}+{\text{θ}}_{2}+\ldots =\overset{\infty }{\underset{i=0}{\sum }}\;{\text{θ}}_{i}$$

and the total areal density of H_2_O molecules adsorbed is:

(A9) $$N_{H2\text{O}}=N_{SA}(0{\text{ θ}}_{0}+1{\text{ θ}}_{1}+2{\text{ θ}}_{2}+\ldots )=N_{SA}\overset{\infty }{\underset{i=0}{\sum }}\;i\;{\text{θ}}_{i}$$

In a dynamic situation, as in our humidity sensing tests, the numbers of molecules in each site change via adsorption and desorption processes via interactions with H_2_O molecules in the gas phase. In each of these processes, an adsorption site initially in the coverage fraction ${\text{θ}}_{i}$ either changes into fraction ${\text{θ}}_{i+1}$ or fraction ${\text{θ}}_{i-1}$, respectively. These exchange processes are visualized in Figure A1 for the three cases of zero, one and two layers already adsorbed.

Mathematically, these exchange processes can be described by a system coupled differential equations:

(A10) $$\left(\begin{array}{c} \frac{d\;\theta_{0}(t)}{dt}\\ \frac{d\;\theta_{1}(t)}{dt}\\ \frac{d\;\theta_{2}(t)}{dt}\\ \frac{d\;\theta_{3}(t)}{dt}\\ \frac{d\;\theta_{4}(t)}{dt}\\ \vdots \end{array})=(\begin{array}{cccccc} -\alpha \;{\text{p}}_{A}(t) & +\beta & 0 & 0 & 0 & \ldots \\ +\alpha \;{\text{p}}_{A}(t) & -\beta -\gamma \;{\text{p}}_{A}(t) & +\delta & 0 & 0 & \ldots \\ 0 & +\gamma \;{\text{p}}_{A}(t) & -\delta -\gamma \;{\text{p}}_{A}(t) & +\delta & 0 & \ldots \\ 0 & 0 & +\gamma \;{\text{p}}_{A}(t) & -\delta -\gamma \;{\text{p}}_{A}(t) & +\delta & \ldots \\ 0 & 0 & 0 & +\gamma \;{\text{p}}_{A}(t) & -\delta -\gamma \;{\text{p}}_{A}(t) & \ddots \\ \vdots & \vdots & \vdots & \vdots & \ddots & \ddots \end{array})(\begin{array}{c} \theta_{0}(t)\\ \theta_{1}(t)\\ \theta_{2}(t)\\ \theta_{3}(t)\\ \theta_{4}(t)\\ \vdots \end{array}\right)$$

The first equation in this set describes the evolution of empty adsorption sites directly on the adsorbent surface. The second describes the evolution of singly occupied sites on the adsorbate surface. This equation is the same as the Langmuir kinetic Equation (A7), except for the two terms involving the parameters γ and δ, which account for the possibility of adsorbing and desorbing a first on-top layer All following kinetic equations describe adsorption/desorption processes on top-on layers i and exchange processes between layers i and i−1 and i+1, respectively.

Equations (A7) and (A10) have been numerically solved using a MATHEMATICA program. With the solutions for the individual ${\text{θ}}_{i}$ the number densities of adsorbed H_2_O molecules were evaluated using Equation (A9). As suggested by Equation (A1), this latter number was fitted to the experimentally observed sensor resistance changes by involving a suitably chosen proportionality constant.

## References

[1] Zhao J., Huo L.-H., Gao S., Zhao H., Zhao J.-G. (2006). Alcohols and acetone sensing properties of SnO_2_ thin films deposited by dip-coating. Sens. Actuators B Chem..

[2] Wang H.C., Li Y., Yang M.J. (2006). Fast response thin film SnO_2_ gas sensors operating at room temperature. Sens. Actuators B Chem..

[3] Anothainart K., Burgmair M., Karthigeyan A., Zimmer M., Eisele I. (2003). Light enhanced NO_2_ gas sensing with tin oxide at room temperature: Conductance and work function measurements. Sens. Actuators B Chem..

[4] Wei B.-Y., Hsu M.-C., Su P.-G., Lin H.-M., Wu R.-J., Lai H.-J. (2004). A novel SnO_2_ gas sensor doped with carbon nanotubes operating at room temperature. Sens. Actuators B Chem..

[5] Comini E. (2006). Metal oxide nano-crystals for gas sensing. Anal. Chim. Acta.

[6] Suchea M., Katsarakis N., Christoulakis S., Nikolopoulou S., Kiriakidis G. (2006). Low temperature indium oxide gas sensors. Sens. Actuators B Chem..

[7] Afshar M., Preiß E.M., Sauerwald T., Rodner M., Feili D., Straub M., König K., Schütze A., Seidel H. (2015). Indium-tin-oxide single-nanowire gas sensor fabricated via laser writing and subsequent etching. Sens. Actuators B Chem..

[8] Comini E., Cristalli A., Faglia G., Sberveglieri G. (2000). Light enhanced gas sensing properties of indium oxide and tin dioxide sensors. Sens. Actuators B Chem..

[9] Comini E., Faglia G., Sberveglieri G. (2001). UV light activation of tin oxide thin films for NO_2_ sensing at low temperatures. Sens. Actuators B Chem..

[10] Yang T.-Y., Lin H.-M., Wei B.-Y., Wu C.-Y., Lin C.-K. (2003). UV enhancement of the gas sensing properties of nano-TiO_2_. Rev. Adv. Mater. Sci..

[11] Mishra S., Ghanshyam C., Ram N., Bajpai R.P., Bedi R.K. (2004). Detection mechanism of metal oxide gas sensor under UV radiation. Sens. Actuators B Chem..

[12] Fu T. (2007). Sensing properties and mechanism of gas sensor for H_2_S and NO_2_ based on [Cu_5_(bipyO_2_)_6_Cl_8_]Cl_2_. Sens. Actuators B Chem..

[13] Helwig A., Müller G., Eickhoff M., Sberveglieri G. (2007). Dissociative Gas Sensing at Metal Oxide Surfaces. IEEE Sens. J..

[14] Schalwig J., Müller G., Ambacher O., Stutzmann M. (2001). Group-III-nitride based gas sensing devices. Phys. Status Solidi.

[15] Schalwig J., Müller G., Eickhoff M., Ambacher O., Stutzmann M. (2002). Gas sensitive GaN/AlGaN-heterostructures. Sens. Actuators B Chem..

[16] Stutzmann M., Steinhoff G., Eickhoff M., Ambacher O., Nebel C.E., Schalwig J., Neuberger R., Müller G. (2002). GaN-based heterostructures for sensor applications. Diam. Relat. Mater..

[17] Offermans P., Vitushinsky R., Fabrication A.D. (2013). NO2 detection with AlGaN/GaN 2DEG channels for air quality monitoring. IEEE Sens. J..

[18] Vitushinsky R., Crego-Calama M., Brongersma S.H., Offermans P. (2013). Enhanced detection of NO2 with recessed AlGaN/GaN open gate structures. Appl. Phys. Lett..

[19] Pearton S.J., Kang B.S., Kim S., Ren F., Gila B.P., Abernathy C.R., Lin J., Chu S.N.G. (2004). GaN-based diodes and transistors for chemical, gas, biological and pressure sensing. J. Phys. Condens. Matter.

[20] Paul S., Helwig A., Müller G., Furtmayr F., Teubert J., Eickhoff M., Sumit P. (2012). Opto-chemical sensor system for the detection of H_2_ and hydrocarbons based on InGaN/GaN nanowires. Sens. Actuators B Chem..

[21] Teubert J., Paul S., Helwig A., Müller G., Eickhoff M., Kohl C.-D., Wagner T. (2014). Group III-Nitride Chemical Nanosensors with Optical Readout. Gas Sensing Fundamentals.

[22] Maier K., Helwig A., Müller G., Becker P., Hille P., Schörmann J., Teubert J., Eickhoff M. (2014). Detection of oxidising gases using an optochemical sensor system based on GaN/InGaN nanowires. Sens. Actuators B Chem..

[23] Weidemann O., Hermann M., Steinhoff G., Wingbrant H., Lloyd Spetz A., Stutzmann M., Eickhoff M. (2003). Influence of surface oxides on hydrogen-sensitive Pd:GaN Schottky diodes. Appl. Phys. Lett..

[24] Steinhoff G., Hermann M., Schaff W.J., Eastman L.F., Stutzmann M., Eickhoff M. (2003). pH response of GaN surfaces and its application for pH-sensitive field-effect transistors. Appl. Phys. Lett..

[25] Eickhoff M., Neuberger R., Steinhoff G., Ambacher O., Müller G., Stutzmann M. (2001). Wetting Behaviour of GaN Surfaces with Ga- or N-Face Polarity. Phys. Status Solidi.

[26] Tan O.Z., Tsai K.H., Wu M.C.H., Kuo J.L. (2011). Structural and dynamic properties of water on the GaN polar surface. J. Phys. Chem. C.

[27] Bermudez V.M., Long J.P. (2000). Chemisorption of H_2_O on GaN(0001). Surf. Sci..

[28] Coan M.R., León-Plata P., Seminario J.M. (2012). Ab Initio Analysis of the Interactions of GaN Clusters with Oxygen and Water. J. Phys. Chem. C.

[29] Helwig A., Müller G., Garrido J.A., Eickhoff M. (2008). Gas sensing properties of hydrogen-terminated diamond. Sens. Actuators B Chem..

[30] Beer S., Helwig A., Müller G., Garrido J., Stutzmann M. (2013). Water adsorbate mediated accumulation gas sensing at hydrogenated diamond surfaces. Sens. Actuators B Chem..

[31] Krstev I., Helwig A., Müller G., Garrido J., Stutzmann M. (2014). Detection of random vapour concentrations using an integrating diamond gas sensor. Sens. Actuators B Chem..

[32] Groß A., Beulertz G., Marr I., Kubinski D.J., Visser J.H., Moos R. (2012). Dual mode NO_x_ sensor: Measuring both the accumulated amount and instantaneous level at low concentrations. Sensors. (Basel).

[33] Marr I., Groß A., Moos R. (2014). Overview on conductometric solid-state gas dosimeters. J. Sens. Sens. Syst..

[34] Landstrass M.I., Ravi K.V. (1989). Hydrogen passivation of electrically active defects in diamond. Appl. Phys. Lett..

[35] Maki T., Shikama S., Komori M., Sakaguchi Y., Sakuta K., Kobayashi T. (1992). Hydrogenating Effect of Single-Crystal Diamond Surface. Jpn. J. Appl. Phys..

[36] Hokazono A., Kawarada H. (1997). Enhancement/depletion surface channel field effect transistors of diamond and their logic circuits. Jpn. J. Appl. Phys..

[37] Hayashi K., Yamanaka S., Okushi H., Kajimura K. (1996). Study of the effect of hydrogen on transport properties in chemical vapor deposited diamond films by Hall measurements. Appl. Phys. Lett..

[38] Chakrapani V., Angus J.C., Anderson A.B., Wolter S.D., Stoner B.R., Sumanasekera G.U. (2007). Charge transfer equilibria between diamond and an aqueous oxygen electrochemical redox couple. Science.

[39] Kromka A., Davydova M., Rezek B., Vanecek M., Stuchlik M., Exnar P., Kalbac M. (2010). Gas sensing properties of nanocrystalline diamond films. Diam. Relat. Mater..

[40] Davydova M., Stuchlik M., Rezek B., Kromka A. (2012). Temperature enhanced gas sensing properties of diamond films. Vacuum.

[41] Maier F., Riedel M., Mantel B., Ristein J., Ley L. (2000). Origin of surface conductivity in diamond. Phys. Rev. Lett..

[42] Helwig A., Müller G., Sberveglieri G., Eickhoff M. (2009). On the low-temperature response of semiconductor gas sensors. J. Sens..

[43] Fiz R., Hernandez-Ramirez F., Fischer T., Lopez-Conesa L., Estrade S., Peiro F., Mathur S. (2013). Synthesis, characterization, and humidity detection properties of Nb_2_O_5_ nanorods and SnO_2_/Nb_2_O_5_ heterostructures. J. Phys. Chem. C.

[44] Maier K., Müller G., Teubert J., Eickhoff M. (2015). H_2_O adsorption at luminescent InGaN nanowires. Sens. Actuators B Chem..

[45] Helwig A., Müller G., Sberveglieri G., Faglia G. (2007). Gas sensing properties of hydrogenated amorphous silicon films. IEEE Sens. J..

[46] Brunauer S., Emmett P.H., Teller E. (1938). Adsorption of Gases in Multimolecular Layers. J. Am. Chem. Soc..

[47] Henzler M., Göpel W. (1994). Oberflächenphysik des Festkörpers.

[48] Lueth H. (2010). Solid Surfaces, Interfaces and Thin Films.

[49] Thomson W.F.R.S. (1871). LX. On the equilibrium of vapour at a curved surface of liquid. Philos. Mag. Ser. 4.

[50] Moore W.J. (1990). Grundlagen der Physikalischen Chemie.

[51] Galvin K.P. (2005). A conceptually simple derivation of the Kelvin equation. Chem. Eng. Sci..

[52] Randeniya L.K., Shi H., Barnard A.S., Fang J., Martin P.J., Ostrikov K.K. (2013). Harnessing the influence of reactive edges and defects of graphene substrates for achieving complete cycle of room-temperature molecular sensing. Small.

[53] Hellmich W., Bosch-v. Braunmühl C., Müller G., Sberveglieri G., Berti M., Perego C. (1995). The kinetics of formation of gas-sensitive RGTO-SnO_2_ films. Thin Solid Films.

[54] Sberveglieri G., Faglia G., Groppelli S., Nelli P. RGTO: A New Technique for Preparing SnO_2_ Sputtered Thin Film As Gas Sensors. Proceedings of the TRANSDUCERS’91: 1991 International Conference on Solid-State Sensors and Actuators.

